# Hypoxia-induced epithelial-mesenchymal transition and fibrosis for the development of breast capsular contracture

**DOI:** 10.1038/s41598-019-46439-7

**Published:** 2019-07-16

**Authors:** Yao-Lung Kuo, I-Ming Jou, Seng-Feng Jeng, Chun-Hui Chu, Jhy-Shrian Huang, Tai-I Hsu, Li-Ren Chang, Po-Wei Huang, Jian-An Chen, Ting-Mao Chou

**Affiliations:** 1 Department of Surgery, National Cheng Kung University Hospital, College of Medicine, National Cheng Kung University, Tainan and Dou-Liou, Dou-Liou, Taiwan; 2 Department of Orthopaedics, E-DA Hospital, Kaohsiung, Taiwan; 3 Department of Plastic Surgery, E-DA Hospital, Kaohsiung, Taiwan; 4 Charites Plastic Surgery Clinic, Tainan, Taiwan; 5 R&D Division, Maxigen Biotech Inc., Taoyuan City, Taiwan; 6 Department of Orthopaedics, College of Medicine, National Cheng Kung University, Tainan, Taiwan; 7 Division of Urology, Department of Surgery, Zuoying Branch of Kaohsiung Armed Forces General Hospital, Kaohsiung, Taiwan; 8 Department of Plastic and Reconstructive Surgery, E-Da Hospital, Kaohsiung, Taiwan; 9 Department of Plastic Surgery, E-Da Cancer Hospital, Kaohsiung, Taiwan

**Keywords:** Mechanisms of disease, Molecular medicine

## Abstract

Fibrosis has been considered as a major cause of capsular contracture. Hypoxia has widely emerged as one of the driving factors for fibrotic diseases. The aim of this study was to examine the association between hypoxia-induced fibrosis and breast capsular contracture formation. Fibrosis, epithelial-mesenchymal transition (EMT), expression levels of hypoxia-inducible factor-1α (HIF-1α), vimentin, fibronectin, and matrix metalloproteinase-9 (MMP-9) in tissues from patients with capsular contracture were determined according to the Baker classification system. Normal breast skin cells in patients with capsular contracture after implant-based breast surgery and NIH3T3 mouse fibroblasts were cultured with cobalt chloride (CoCl_2_) to mimic hypoxic conditions. Treatment responses were determined by detecting the expression of HIF-1α, vimentin, fibronectin, N-cadherin, snail, twist, occludin, MMP-9, tissue inhibitor of metalloproteinase-1 (TIMP-1) and -2, as well as phosphorylated ERK. The expression levels of HIF-1α, vimentin, fibronectin, and fibrosis as well as EMT were positively correlated with the severity of capsular contracture. MMP-9 expression was negatively correlated the Baker score. Hypoxia up-regulated the expression of HIF-1α, vimentin, fibronectin, N-cadherin, snail, twist, TIMP-1 and -2, as well as phosphorylated ERK in normal breast skin cells and NIH3T3. Nonetheless, the expression levels of MMP-9 and occludin were down-regulated in response to CoCl_2_ treatment. This study is the first to demonstrate the association of hypoxia-induced fibrosis and capsular contracture.

## Introduction

Among all cosmetic procedures, implant-based breast surgery is one of the most commonly performed ones^1^. The most widely used implants nowadays are silicon-enveloped implants. It can be either a silicone outer shell with silicone gel filling, or a silicone outer shell filled with saline solution^2,3^. No matter what kind of implant is inserted into patient’s breast, it inevitably induces a foreign body reaction. This reaction will lead to an excessive fibrotic process, which eventually results in a common complication following implant-based breast surgeries, designated capsular contracture^4^. This complication causes painful and deformed breasts. Reoperation is usually required if the situation gets worse. However, the underlying mechanisms regarding the pathogenesis of capsular contracture remain largely unclear.

Traditionally, capsular contracture can be classified using the Baker classification system and divided into four classes. It has been documented that fibroblasts, which produce collagen, accumulate at the contact region of the capsule and implant. The number of fibroblasts is positively correlated with the Baker grade^5^. Further histological examinations reveal that the capsule tissue is composed of uniformly distributed collagen fibers in which the orientation and organization are supposed to be altered as the disease severity becomes worse. The fibers become thicker and cable-like bundles which orientate themselves perpendicular to the fibroblasts to produce a helical orientation as the severity progresses^6^. Furthermore, activated tissue-resident fibroblasts can convert into myofibroblasts, Myofibroblasts are a group of contractile fibroblasts which provide a contractile force to decrease the surface area of the capsule while the collagen matrix remodels and stabilizes the contracture. In the fibrotic condition, myofibroblasts persistently proliferate and are resistant to apoptosis, leading to assembly of extracellular matrix (ECM) with subsequent dysfunction of the target organ^7,8^. Epithelial-mesenchymal transition (EMT) has been accepted as the predominant source of myofibroblast and plays a critical role in the development of organ fibrosis, as the matrix-producing myofibroblasts can arise from cells of the epithelial lineage in response to injuries^9–11^. Therefore, identification of the detailed mechanisms driving fibrotic process is important in understanding the pathophysiology of capsular contracture.

Hypoxia has widely emerged as one of the driving factors for fibrogenesis. Activation of hypoxia-inducible factor-1 (HIF-1) in renal epithelial cells under hypoxic conditions induces fibrogenesis by increasing the expression of ECM-associated factors and EMT^12^. Moreover, hypoxia via HIF-1α contributes to the development and progression of pulmonary fibrosis through production of profibrotic factors HIF1α up-regulates the ADORA2B receptor on alternatively activated macrophages and contributes to pulmonary fibrosis^13^. Therefore, targeting HIF-1α should be an ideal therapeutic approach in treating fibrosis^14^. Because the capsules are usually located between normal tissues and the avascular artificial prosthesis, they can encounter relatively hypoxic conditions when compared to normal tissues. Therefore, it would be intriguing to decipher if the hypoxia-induced fibrosis is associated with the formation of capsular contracture.

In this study, we first demonstrated that the expression of HIF-1α and its downstream regulators responsible for fibrogenesis were contiminantly up-regulated in patients with capsular contracture. Further *in vitro* experiments reveal that hypoxia drives the fibrotic process in primary breast skin cells and NIH3T3 mouse fibroblasts by determining the levels of mesenchymal markers, including vimentin, fibronectin, and N-cadherin in response to cobalt chloride (CoCl_2_) stimulation. Furthermore, cytoskeletal disorganization can also be observed in tissues and cells under hypoxic conditions, implicating that hypoxia may not only drive fibrosis, but disturb the homeostasis of ECM in capsular contracture.

## Results

### Disorganization of the extracellular matrix (ecm) in breast capsule contracture

**Figure 1:**
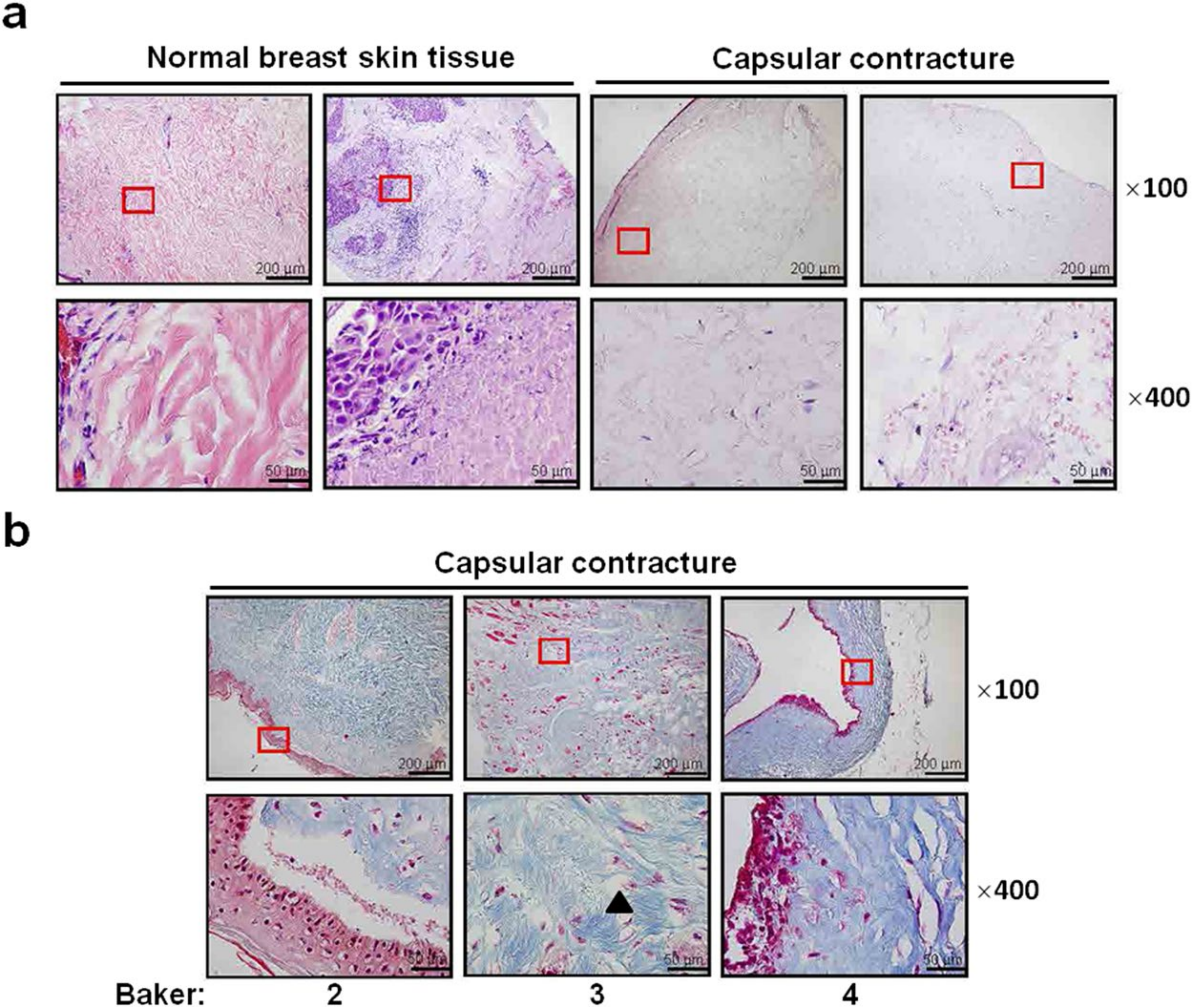
Extracellular matrix (ECM) structure in tissue from patients with capsular contracture. (**a**) Organized and compact ECM in interstitial space from normal breast skin tissues compared with capsular contracture specimens (Baker = 2), as determined by hematoxylin and eosin (H&E) staining. (**b**) Expression of collagen fiber in capsular contracture, as assessed by Masson’s Trichrome staining. Arrow head, cord-like pattern. Bars shown at ×100 and ×400 magnification correspond to 200 μm and 50 μm, respectively. Baker: Baker classification.

To compare the ECM structure in patients with capsular contracture after implant-based breast surgeries, histological examinations were performed on samples collected from patients with capsulectomy and scar excision surgery. The control group is the normal skin that were included during scar excision. Organized ECM with fiber tissues surrounding the skin cells was observed in the control group compared with those in the breast capsule tissue, which demonstrated loosened ECM, as shown by H&E examinations (Fig. 1a). Furthermore, “cord-like” pattern of collagen fibers can be identified in the capsule specimens, as demonstrated by Masson’s Trichrome staining (Fig. 1b, arrow head).

### Increased expression of HIF-1α, vimentin, fibronectin, but decreased levels of matrix metallo-proteinase-9 (MMP-9) in breast capsular contracture

**Figure 2:**
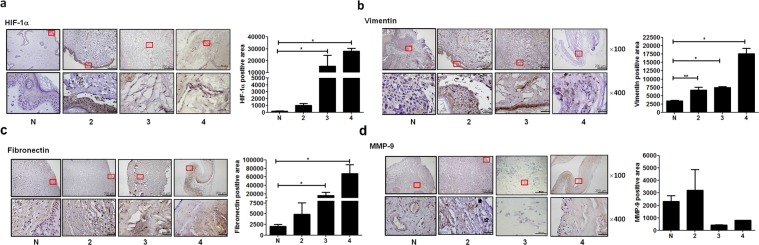
Expression of hypoxia inducible factor-1α (HIF-1α), vimentin, fibronectin and matrix metalloproteinase-9 (MMP-9) in tissue from patients with breast capsular contracture. Immunohistochemical staining and quantitative analyses of (**a**) HIF-1α (**b**) vimentin (**c**) fibronectin and (**d**) MMP-9 in thicken tissue from five (Baker = 2), three (Baker = 3), and three (Baker = 4) breast capsules patients with capsular contracture which were sorted by the Baker classification system. Four normal skin (N) were included during scar excision in patients with capsular contracture after implant-based breast surgery. (Bars shown at ×100 and ×400 magnification correspond to 200 μm and 50 μm, respectively. Arrow, up-regulated MMP-9 expression in relatively loose area of ECM; Hollow arrow, down-regulated MMP-9 expression in relatively dense area of ECM; Asterisk: up-regulated fibronectin expression in relatively dense area of ECM. Values are the mean ± SEM. *p < 0.05. See Fig. 1 for other definitions.

To link tissue hypoxia to fibrosis in breast capsules, immunohistochemical staining was performed to examine the expression of HIF-1α, vimentin, fibronectin, and MMP-9. We showed higher expression of HIF-1α, vimentin, and fibronectin in capsule tissues from patients (Baker = 2, 3, and 4) when compared to normal control (N) (Fig. 2a–c). In particular, reduced expression of MMP-9 can be identified in capsule tissues from patients with severe symptoms (Baker = 3 and 4) (Fig. 2d). Moreover, the expression of MMP-9 was up-regulated in the loose region of ECM (Fig. 2d, arrow) whereas MMP-9 levels were reduced in the relatively dense region (Fig. 2d, hollow arrow). The expression pattern of fibronectin was in contrast to that of MMP-9. Fibronectin expression was down-regulated in the loose region, but up-regulated in the dense region (Fig. 2c, asterisk). These results suggest that HIF-1α and its downstream regulators are responsible for the fibrotic process and ECM remodeling in the development of breast capsular contracture.

### Hypoxia induces fibrogenic activity in primary breast skin cells and mouse fibroblasts by activating EMT and ECM remodeling

**Figure 3:**
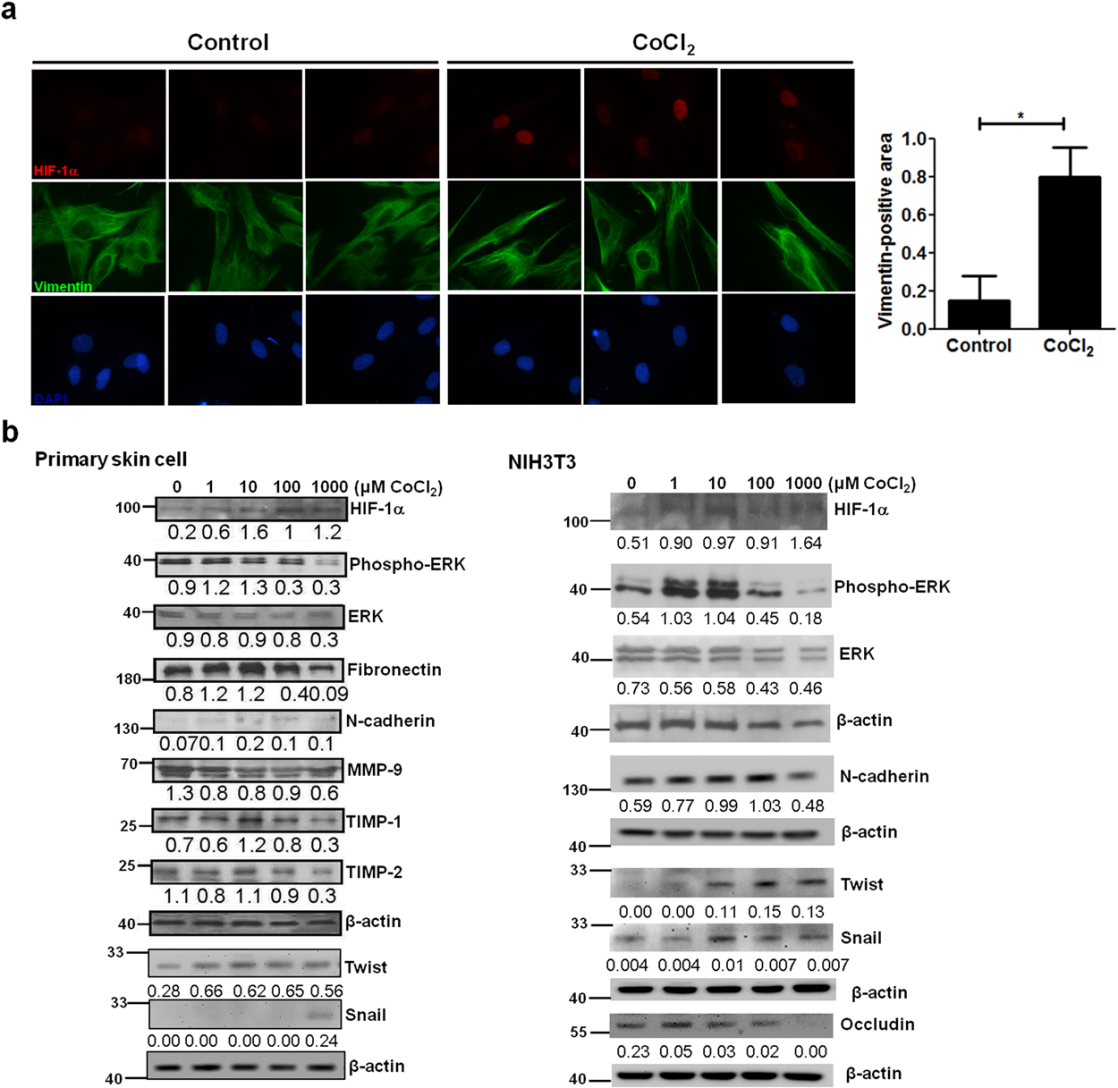
Expression of HIF-1α, epithelial and mesenchymal markers, and ECM remodeling molecules in primary breast skin cells and fibroblasts under hypoxic induction. Normal skin were included during scar excision in patients with capsular contracture after implant-based breast surgery. Primary breast skin cells and NIH3T3 mouse fibroblasts were treated with cobalt chloride (CoCl_2_ to mimic hypoxia conditions). (**a**) Immunofluorescence staining of HIF-1α (Alexa Fluor 594 stained [red]), vimentin (Alexa Fluor 488 stained [green]) and nucleus (DAPI stained [blue]) in primary breast skin cells treated with 0 (Control) and 1 mM of CoCl_2_. Figures were shown at ×1000 magnification. Further quantification was measured by ImageJ software. (**b**) Expression of HIF-1α, phospho-ERK, ERK, mesenchymal markers, fibronectin, N-cadherin, twist, and snail, and an epithelial marker, occludin, as well as ECM remodeling molecules, MMP-9, tissue inhibitor of metalloproteinase-1 (TIMP-1) and -2 in primary breast skin cells and NIH3T3 treated with increasing concentrations (1 μM to 1 mM) of CoCl_2_, as determined by immunoblotting. Quantitation of intensity of the bands corresponding to the indicated proteins compared with β-actin in cell extracts. Values are the mean ± SEM. *p < 0.05.

To further clarify the role of hypoxia for fibrogenic activity at the molecular level, primary breast skin cells were subjected to the hypoxic condition by culturing with CoCl_2_. Hypoxia augmented HIF-1α expression in accordance with increased levels of vimentin, as demonstrated by immunofluorescence and quantitative analyses (Fig. 3a). Furthermore, the expression of HIF-1α was up-regulated in primary breast skin cells and NIH3T3 in response to CoCl_2_ treatment dose-dependently. Meanwhile, the expression levels of mesenchymal markers including fibronectin, N-cadherin, twist, and snail, the ECM remodeling molecules, tissue inhibitor of metalloproteinase-1 (TIMP-1) and -2, as well as the signaling transducer phosphorylated ERK were concomitantly increased in these cells under hypoxic culture, as determined by immunoblot analysis. Interestingly, the expression levels of MMP-9 and occludin were down-regulated in response to CoCl_2_ treatment (Fig. 3b).

## Discussion

Fibroitic condition is a hypoxia-related disease in which the excessive accumulation of ECM, including fibronectin, collagens, and vimentin has been identified. Compelling evidence indicate a key role of HIF-1α in activated fibroblasts which contribute to the up-regulated gene expression of ECM. Furthermore, epithelial mesenchymal transition (EMT) can be recognized by acquisition of mesenchymal markers during fibrogrnesis. EMT produces more ECM-related proteins such as α-SMA and vimentin and requires HIF-1α expression^15–17^. According to the histological and immunohistochemical findings in our study (Figs 1 and 2), we observed that HIF-1ɑ and its downstream regulators, including vimentin, and fibronectin, as well as fibrosis were expressed at higher levels in breast capsules from patients with severe symptoms (Baker = 4) in comparison to those with mild ones (Baker = 2). These findings indicate that HIF-1α expression may promote capsule formation by facilitating EMT or fibrosis. To our knowledge, this study is the first to link tissue hypoxia to breast capsular contracture.

It is generally accepted that patients with capsular fibrosis have a reduced MMP-to-TIMP ratio that correlates with the Baker classification system^18^. The decrease in this ratio can cause increased synthesis and deposition of ECM-related molecules surrounding alloplastic breast implants, leading to a profibrotic state^19^. Interestingly, our cell and tissue studies mutually demonstrated that hypoxia down-regulated the expression of MMP-9 in primary breast skin cells and capsular tissue sections (Figs 2d and 3b). In contrast, there were up-regulated levels of TIMP-1 and -2 in cell upon hypoxic induction (Fig. 3b). Therefore, the decrease in MMP-9 to TIMP-1 and -2 ratio may explain the enhanced expression of fibronection, N-cadherin, and vimentin in hypoxia-induced cells and capsule tissues.

The signaling pathways through ERK activation have been shown to mediate hypoxia-induced EMT in various kinds of fibrotic diseases^20–22^. In our results, we demonstrated that the expression of phosphorylated ERK was increased in primary breast skin cells upon hypoxic induction (Fig. 3). Interestingly, Nonetheless, further inhibition of ERK activity should be applied to assign causality to the hypoxia-induced expression of ECM remodeling molecules in our cell study. Besides, there are limitations to the present work. For instance, a knockout or knockdown system for HIF-1α has to be performed in an animal model of capsular contracture to clearly examine the role in association with capsule fibrosis, which requires further investigations.

In conclusion, with the analyses of the breast capsule specimens and human primary skin cells as well as mouse fibroblasts, we demonstrate that hypoxia is responsible for capsular fibrosis and ECM deposition, attributing to the reduced ratio of MMP-to-TIMP. These findings may contribute to the development of pharmacological therapies targeting HIF-1α signaling pathways for the treatment of breast capsular contracture.

## Methods

### Ethics statement

Informed consent was obtained from all patients prior to sample collection. This study was approved and all methods were performed in accordance with the relevant guidelines and regulations by the Institutional Review Board of E-DA Hospital. Normal skin from four individuals (n = 4) and breast capsules from seven patients (n = 11, Baker score = 2 (5), Baker score = 3 (3), and Baker score = 4 (3) were included in this study.

### Primary culture of normal breast skin cells

Normal skin were included during scar excision in patients with capsular contracture after implant-based breast surgery. Skin samples were treated with trypsin (0.05%) for 5 min. Primary breast skin cells were cultured in Dulbecco’s Modified Eagle Medium (ThermoFisher) supplied with 10% fetal bovine albumin.

### Masson’s trichrome staining, histopathological and immunohistochemical examinations

Human breast capsules from patients were collected during scar excision surgery. Hematoxylin and eosin-stained paraffin-embedded breast capsule sections were prepared and classified using the Baker classification system. Briefly, class 1 and 2 are not clinically significant, in that class 1 describes a breast that looks and feels absolutely natural and class 2 describes a breast with minimal contracture in that the surgeon can tell surgery has been performed but there are no symptoms. Class 3 and 4 are clinically significant and symptomatic, with class 3 describing moderate contracture with some firmness felt by the patient and class 4 describing severe contracture which is obvious from observation and symptomatic in the patients. For immunohistochemical staining, normal skin and breast capsule samples from the patients were snap-frozen and embedded in paraffin. The sections were deparaffinized in xylene, dehydrated in alcohol, epitope unmasked by heating, washed with H_2_O_2_ in PBS, and stained with antibodies against HIF-1α (Genetex), vimentin (BD Biosciences), fibronectin (Genetex), and MMP-9 (Millipore) in combination with the chromogen 3-amino-9-ethylcarbazole (Zymed). The deparaffinized sections were subjected to Masson’s Trichrome staining according to manufacturer’s instructions (Sigma-Aldrich). Positive areas were quantitated by averaging their signals in 3 randomly selected fields at the ×400 magnification. The signal intensity was quantitated using ImageJ software (National Institutes of Health).

### Immunoblot and immunofluorescence assessments

NIH3T3 and primary breast skin cells were treated with 1 μM to 1 mM concentrations of CoCl_2_ to mimic hypoxic conditions. Cell lysates were subjected to immunoblot analyses with antibodies against HIF-1α (Genetex), total ERK (Abcam), phosphorylated ERK (Abcam), vimentin (BD Biosciences), fibronectin (Genetex), N-cadherin (Genetex), occludin (Abcam), MMP-9 (Millipore), snail (Cell Signaling), twist (Sigma-Aldrich), TIMP-1 (Epitomics) and TIMP-2 (Millipore) in combination with a horseradish peroxidase-conjugated secondary antibody (Jackson ImmunoResearch) and quantitative control anti-β-actin antibodies (Millipore). For immunofluorescence staining, NIH3T3 cells were subjected to hypoxic induction and stained with antibodies against HIF-1α (Genetex) and vimentin (BD Biosciences), followed by Alexa Fluor 488- and Alexa Fluor 594-conjugated secondary antibodies (Invitrogen), respectively. The signal intensity was further quantitated using ImageJ software (National Institutes of Health). Quantitation of intensity of the bands corresponding to the indicated proteins compared with β-actin in cell extracts.

### Statistical analysis

Data are expressed as mean ± SEM. Statistical significance between two groups was assessed using Student’s *t* test. The correlation among HIF-α, Vimentin, Fibronectin, and MMP-9 expression and the different histological grades of capsular tissues were analyzed using the Spearman correlation rank test. P values less than 0.05 were considered significant.

## Supplementary information

full-length gels and blots
