# Supplementary material for: Hypoxia-induced epithelial-mesenchymal transition and fibrosis for the development of breast capsular contracture
Source: Sci Rep. 2019 Jul 16;9:10269. doi: 10.1038/s41598-019-46439-7 (PMC6635377; doi:10.1038/s41598-019-46439-7)

Hypoxia-induced epithelial-mesenchymal transition and fibrosis for the development of breast capsular contracture

Yao-Lung Kuo[1], I-Ming Jou[2], Seng-Feng Jeng[3], Chun-Hui Chu[4], Jhy-Shrian Huang[5], Tai-I Hsu[6], Li-Ren Chang[3], Po-Wei Huang[7], Jian-An Chen[8] and Ting-Mao Chou[9]

Primary skin cell

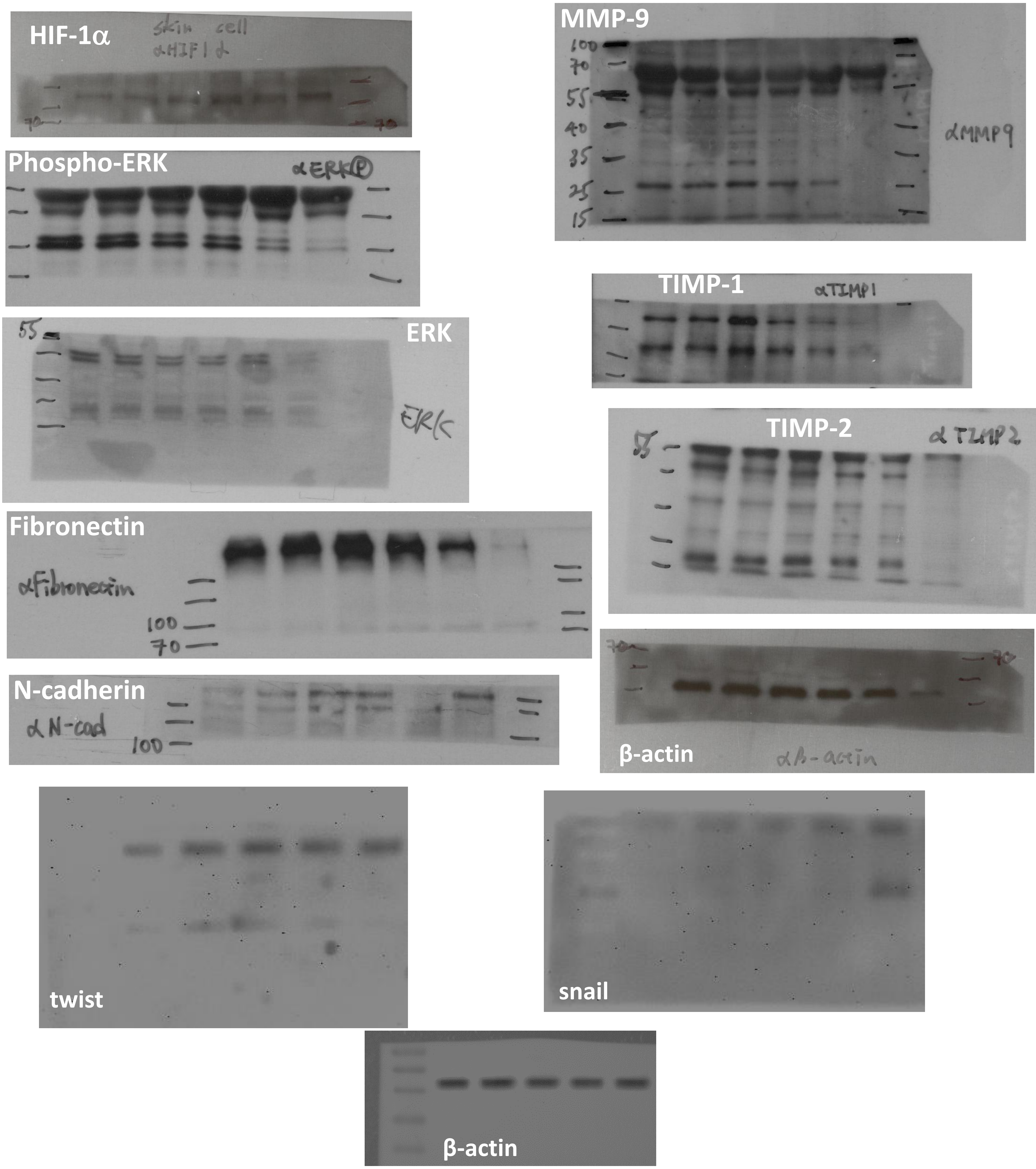

Hypoxia-induced epithelial-mesenchymal transition and fibrosis for the development of breast capsular contracture

Yao-Lung Kuo[1], I-Ming Jou[2], Seng-Feng Jeng[3], Chun-Hui Chu[4], Jhy-Shrian Huang[5], Tai-I Hsu[6], Li-Ren Chang[3], Po-Wei Huang[7], Jian-An Chen[8] and Ting-Mao Chou[9]

NIH3T3

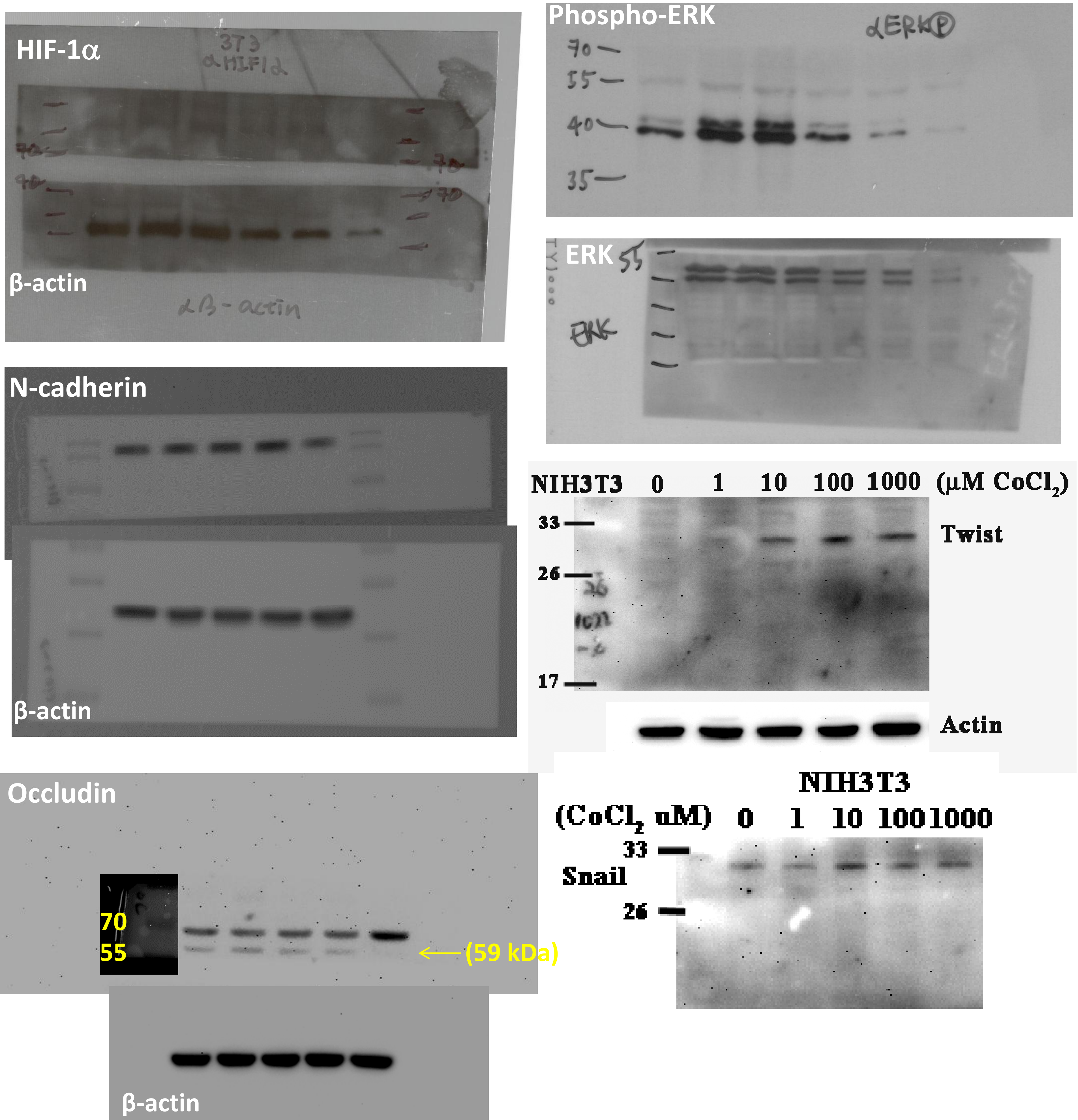

Supplement: Supplementary file 1 — full-length gels and blots [file 41598_2019_46439_MOESM1_ESM.pdf]
